# Anadromous Arctic Char Microbiomes: Bioprospecting in the High Arctic

**DOI:** 10.3389/fbioe.2019.00032

**Published:** 2019-02-26

**Authors:** Erin F. Hamilton, Geraint Element, Peter van Coeverden de Groot, Katja Engel, Josh D. Neufeld, Vishal Shah, Virginia K. Walker

**Affiliations:** ^1^Department of Biology, Queen's University, Kingston, ON, Canada; ^2^Department of Biology, University of Waterloo, Waterloo, ON, Canada; ^3^College of the Sciences and Mathematics, West Chester University, West Chester, PA, United States

**Keywords:** Arctic char, salmonid fish, anadromous, microbiomes, bioprospecting, aquaculture, Arctic Ocean, aquatic biotechnology

## Abstract

Northern populations of Arctic char (*Salvelinus alpinus*) can be anadromous, migrating annually from the ocean to freshwater lakes and rivers in order to escape sub-zero temperatures. Such seasonal behavior demands that these fish and their associated microbiomes adapt to changes in salinity, temperature, and other environmental challenges. We characterized the microbial community composition of anadromous *S. alpinus*, netted by Inuit fishermen at freshwater and seawater fishing sites in the high Arctic, both under ice and in open water. Bacterial profiles were generated by DNA extraction and high-throughput sequencing of PCR-amplified 16S ribosomal RNA genes. Results showed that microbial communities on the skin and intestine of Arctic char were statistically different when sampled from freshwater or saline water sites. This association was tested using hierarchical Ward's linkage clustering, showing eight distinct clusters in each of the skin and intestinal microbiomes, with the clusters reflecting sampling location between fresh and saline environments, confirming a salinity-linked turnover. This analysis also provided evidence for a core composition of skin and intestinal bacteria, with the phyla Proteobacteria, Firmicutes, and Cyanobacteria presenting as major phyla within the skin-associated microbiomes. The intestine-associated microbiome was characterized by unidentified genera from families Fusobacteriaceae, Comamonadaceae, Pseudomonadaceae, and Vibrionaceae. The salinity-linked turnover was further tested through ordinations that showed samples grouping based on environment for both skin- and intestine-associated microbiomes. This finding implies that core microbiomes between fresh and saline conditions could be used to assist in regulating optimal fish health in aquaculture practices. Furthermore, identified taxa from known psychrophiles and with nitrogen cycling properties suggest that there is additional potential for biotechnological applications for fish farm and waste management practices.

## Introduction

Fish carry a mucous layer on their epithelial surfaces that consists of mucins, immunoglobulins, antimicrobial peptides, and commensal bacteria, which serve roles in friction reduction, waste removal, osmoregulation, as well as an early line of defense against pathogens (Esteban and Cerezuela, 2015). In addition, variations in mucous layer microbiome composition occur across different life stages, among different species, and across distinct geographies in amphibians and marine mammals, which are known to share certain microbial species with the surrounding water (Boutin et al., 2013; Apprill et al., 2014; Kueneman et al., 2014; Chiarello et al., 2015). Indeed, in farmed Atlantic salmon (*Salmo salar*), the abundance of certain bacterial phyla in skin- and intestine-associated microbiomes changed depending on the water source (Lokesh and Kiron, 2016; Dehler et al., 2017). Structural changes to the microbiota, however, have not been well-described in wild fish populations. This research is noteworthy because it indicates that deliberate shifts in community structure could potentially hinder the development of dysbiosis.

Arctic char (*Salvelinus alpinus*), a salmonid species, is of particular interest given the increased popularity of the farmed product in temperate regions. Wild *S. alpinus* stocks from high latitude waters with an anadromous life history, such that they spend the winter in freshwater lakes to avoid freezing and subsequently migrate to the more nutrient-rich Arctic sea in the summer, could provide insight into the purported turnover in char microbiomes and be of interest to aquaculture biotechnologists.

Although relatively unexploited commercially, Arctic char stocks in the lower Northwest Passage of the Kitikmeot region of Nunavut, Canada represent an essential subsistence fishery to indigenous Inuit communities. With recent altered sea ice patterns as a consequence of climate change, and the potential for increasing stress of pollutants associated with future industrialization, we considered it important to undertake genomic, demographic, physiological and microbial analyses on these fish populations. At present, the stocks are considered healthy due a general lack of commercial fisheries in this region, as well as the relatively long-life span of individual fish, which has been reported as up to 33 years in our samples. As part of this effort, an assessment of the mucosa-associated microbiomes of the skin and intestines of *S. alpinus* from the area in, and surrounding, King William Island Nunavut, has been undertaken. We further explored whether this increased understanding of the microbial communities could inform future biotechnological applications in the management of commercially farmed fish, in addition to other biotechnologies.

## Materials and Methods

### Study Area

**Figure 1:**
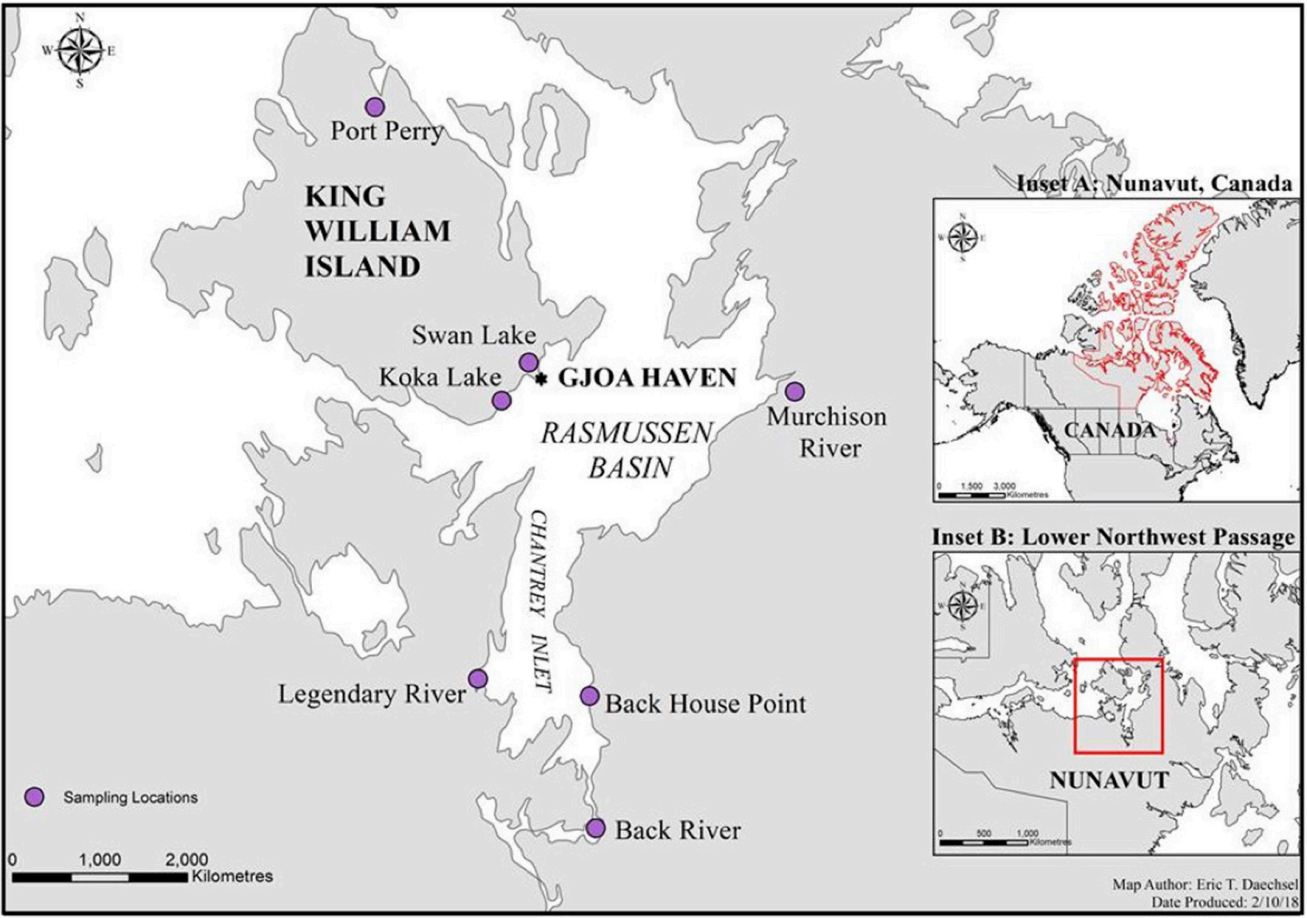
A map of the lower Northwest Passage in Nunavut, Canada and the location of seven distinct fishing sites initially chosen based on Inuit Traditional Ecological Knowledge. The sites fished include five freshwater sites (Port Perry, Swan Lake, Koka Lake, Murchison River, and Back River); and two saltwater sites (Back House Point and Legendary River estuary). Inset A outlines Nunavut, Canada, in red, while inset B showcases the lower Northwest Passage, in red.

Fishing was done within the Kitikmeot region of Nunavut, Canada in the Western Arctic, at seven distinct sites within 200 km of King William Island, located along the lower Northwest Passage (Figure 1; Table 1). Fishing sites were chosen based on Traditional Ecological Knowledge shared by local Inuit elders and in association with the Hunters and Trappers Association of Gjoa Haven, NU, as part of a large-scale fisheries project (Towards; www.arcticfishery.ca). At each fishing site, specific conductance of surface water was obtained with a conductivity meter (Traceable Fisherbrand, Fisher Scientific) to record conductivity and determine our site designations as either freshwater or saline sites (Table 1). The southern region of the study area is unique due to large freshwater influence from major river systems, including Murchison River, Legendary River, Back River, and Hayes River. Therefore, the major sea-water bodies within the region, namely Rasmussen Basin and Chantrey Inlet, are characterized by brackish salinities as defined by Watling (2007), with conductivities between 1,500 and 15,000 μS cm^−1^, diverging from sea salinities more characteristic of the Pacific and Atlantic Oceans (Carmack, 2007). Fishing sites within these locations are referred to as “saline” sites.

**Table 1:** Location and GPS coordinates for each fishing site, followed by designated water source categories and specific conductance measurements shown as conductivity that were determined by a conductivity meter on-site.

**Location and GPS coordinates**	**Water source**	**Conductivity (μS cm^**−1**^)**
Port Perry (N69°33′28.764″, W97°26′13.884″)	Fresh	286
Swan Lake (N68°40′13.62, W95°56′57.408″)	Fresh	880
Koka Lake (N68°32′5.1″, W96°12′45.899″)	Fresh	670
Back House Point (N67°27′27.2″, W95°21′38.6″)	Saline	8,240
Legendary River (N67°31′17.8″, W96°26′21.8″)	Saline	3,450
Murchison River (N68°34′1.2″, W 93°22′37.452″)	Fresh	225
Back River (N66°57′30.70″, W95°18′5.20″)	Fresh	19

### Fish Collection

Commercial (140 mm mesh) and multi-mesh subsistence (5 or 8 panels of 38–140 mm) fishing nets were set during December-June (freshwater lakes under thick ice) and August-September (at open water river estuaries during sea-ice formation and char migration) at distinct geographic sites over a period of 3 years. After setting for several hours, nets were retrieved, and fish were pulled from the nets with nitrile gloves. Using either sterile cotton swabs or an ethanol-sterilized scalpel, the surface mucosal layer of each fish was sampled once per fish with the swabs, or skin scrapings, taken from above the lateral line. The fish were then photographed, weighed, and measured, followed by dissection in an on-site mobile lab. Full intestines were removed using sterile technique, and both skin and intestinal samples were placed in sterile sample tubes or bags, respectively, and frozen at −20°C in a freezer on-site. Samples were shipped frozen and subsequently stored at −20°C until further processing. In addition to the skin and intestine samples, water samples were also taken at each fishing site, in which up to 2 L of water was filtered through sterile 0.22 μm PALL filters in triplicate. The filters were then frozen at −20°C and were subsequently transported and stored at −80°C until further processing.

Fish were sampled in accordance with issued licenses to fish for scientific purposes in the waters of the Northwest Territories, Yukon north slope, and Nunavut (in accordance with section 52 of the general fishery regulations of the fisheries act, Fisheries and Oceans, Canada) along with an associated animal care permit issued by the Fresh Water Institute Animal Care Committee of the Department of Fisheries and Oceans (current permit numbers S-18/19-1045-NU and FWI-ACC AUP-2018-63).

### Sample Processing and DNA Extraction

After initial optimization experimentation to determine the selection of appropriate DNA extraction kits for each sample type, DNA from the skin samples was extracted using a NucleoSpin Soil Extraction Kit (Machery-Nagel, Bethlehem, PA), with modifications following the procedures of Kueneman et al. (2014) in which samples were incubated at 65°C for 10 min before mechanical lysis. In order to maximize yield, the initial lysis step was conducted twice, and double-distilled sterile water was left on the filter for 5 min prior to elution. Extracted DNA concentrations were assessed using an Invitrogen Qubit 4 Fluorometer and a QuantiFluor ONE dsDNA system (Promega Corporation, Madison, WI, USA), followed by qualitative analysis with agarose gel electrophoresis and a NanoDrop One Microvolume spectrophotometer (Thermo Scientific) set to A_260/280_ absorbance. High concentration samples were diluted to 50 ng μL^−1^ prior to PCR amplification, in which starting material for PCR templates ranged from 1 to 50 ng μL^−1^. These skin-associated microbiome samples underwent a pre-amplification PCR step using primers 8F and 1406R (Lane, 1991; Coolen et al., 2005) to amplify the variable V1–V9 region of the bacterial 16S ribosomal RNA (rRNA) gene. The PCR mix for each reaction (50 μL total volume) contained 1X ThermoFisher DreamTaq Buffer (with 2 mM MgCl_2_), 0.4 μM forward and reverse primers, 200 μM dNTPs, 400 ng BSA, 2.5 U ThermoFisher DreamTaq DNA Polymerase, and 2 μL of template. The PCR was performed as follows: 95°C for 5 min, 25 cycles of 95°C for 1 min, 52°C for 1 min, 72°C for 1 min, with a final extension of 72°C for 7 min.

Intestinal samples were partially thawed to excise three slices within the distal intestine (~2 cm from the vent), comprising a total of 5–100 mg epithelial tissue, avoiding feces and connective tissue. The slices were pooled and DNA was extracted using MOBIO UltraClean Tissue and Cells DNA Isolation Kit (QIAGEN Inc., Toronto, ON) following the manufacturer's instructions, except that elution was achieved with double-distilled sterile water rather than EDTA. The DNA concentrations for the intestine-associated microbiome samples were estimated as described for the skin-associated microbiome preparations, but adjusted to 30 ng μL^−1^. Pre-amplified PCR products were also prepared as described above.

In addition, the V4 region of the 16S rRNA gene was amplified on an Illumina sequence platform from DNA isolated from the triplicate water filters using primers 515F and 806R (Caporaso et al., 2011).

### 16S rRNA Gene Sequencing of Mucosal Microbiomes

The V4-V5 region of the 16S rRNA gene was amplified from each of the skin and intestinal amplification products using primers 515F-Y (Parada et al., 2016) and 926R (Quince et al., 2011). Each primer contained a 6-base index sequence for sample multiplexing as well as Illumina flow cell binding and sequencing sites (Bartram et al., 2011). The PCR mix (25 μL total volume) contained 1X ThermoPol Buffer, 0.2 μM forward and reverse primers, 200 μM dNTPs, 15 μg BSA, 0.625 U Hot Start *Taq* DNA polymerase, and 1 μL of template. The PCR was performed as follows: 95°C for 3 min, 35 cycles of 95°C for 30 s, 50°C for 30 s, 68°C for 1 min, and a final extension of 68°C for 7 min. Each amplification reaction was done in triplicate. Equal quantities of each amplicon were pooled. Samples that did not yield a PCR product were not included. No-template controls were added to the Illumina sequencing pool (5 μL), even when amplicons were not detected. Pooled 16S rRNA gene amplicons were subsequently excised from an agarose gel and purified using the Wizard SV Gel and PCR Clean-Up System (Promega, WI, USA). A 5 pM library containing 15% PhiX Control v3 (Illumina Canada Inc, NB, Canada) was sequenced on a MiSeq instrument (Illumina Inc, CA, USA) using a 2 × 250 cycle MiSeq Reagent Kit v2 (Illumina Canada Inc., BC, Canada).

### Sequence Data Analysis, OTU Tables, and Statistics

Sequence reads were demultiplexed using Illumina MiSeq Reporter software version 2.5.0.5. Reads were assembled using the paired-end assembler for Illumina sequences (PANDAseq version 2.8, Masella et al., 2012) with a quality threshold of 0.9, an 8 nucleotide minimum overlap, and 32 nucleotide minimum assembled read length. Assembled reads were analyzed using Quantitative Insights Into Microbial Ecology (QIIME version 1.9.0, Caporaso et al., 2010). Sequences were clustered into operational taxonomic units (OTUs) using UPARSE algorithm USEARCH version 7.0.1090 (Edgar, 2013) at 97% identity and aligned with the Python Nearest Alignment Space Termination tool (PyNAST version 1.2.2, Caporaso et al., 2010). All representative sequences were classified using the Ribosomal Database Project (RDP version 2.2, Wang et al., 2007) with a stringent confidence threshold (0.8) and the Greengenes database (McDonald et al., 2012) was used to assign taxonomy. Chimeric sequences were filtered with UCHIME (Edgar et al., 2011). Before performing statistical analysis, OTUs observed in no-template PCR controls for a sample type were filtered from all samples of that type. OTUs with three or fewer reads within negative PCR controls were retained within the samples, since their low representation in the negative controls rendered them viable representatives in the samples. The OTUs were then rarefied to ~2,000 reads for skin and intestinal microbiome samples. Alpha and beta diversity for samples were analyzed based on rarified OTU tables generated using QIIME with principal coordinate analysis (PCoA). Additional visualizations were done using EMPeror (Vázquez-Baeza et al., 2013). Primer 7 software version 7.0.13 was used for analysis of similarity (ANOSIM) and similarity percentages (SIMPER) analyses (Clarke et al., 2014). Clustering analysis was performed using Statistica 13.0 Academic software.

The hypothesis that skin microbial community composition was linked to salinity was tested by performing hierarchical Ward's linkage clustering (Dillner et al., 2005). Tree cluster analysis was carried out using Ward's method as the amalgamation rule and the distance measured as Euclidean units (Dillner et al., 2005). Using K-means clustering, the samples were divided into K clusters by selecting the number of iterations as 10, and the initial cluster centers chosen to maximize initial between-cluster distances. The output of this step is the list of fish samples that are present in each cluster. The sequence data is publicly available in the European Nucleotide Archive within the European Bioinformatics Institute, under the study: Characterization and Analysis of Skin- and Intestine-associated Microbiomes in the Lower Northwest Passage (Nunavut, Canada) with the following accession number, PRJEB29173.

## Results

### Comparisons of Skin and Intestinal Microbiota Within Individual Fish

**Figure 2:**
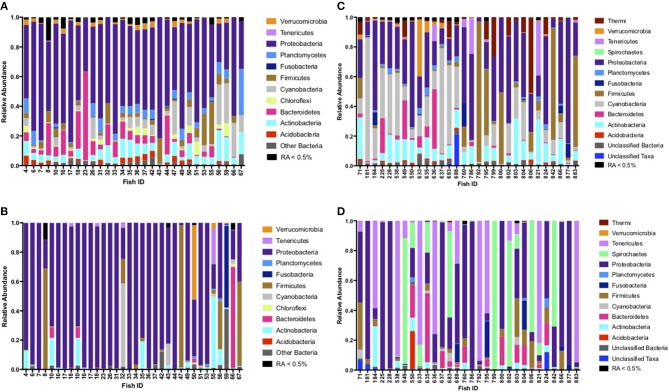
Phyla comparison of skin and intestinal complementary microbiomes for individual fish, shown for samples collected at **(A)** freshwater sites Port Perry, Swan Lake, and Koka Lake for skin-associated microbiomes, **(B)** freshwater sites Port Perry, Swan Lake, and Koka Lake for intestine-associated microbiomes, **(C)** saltwater sites, Back House Point and Legendary River for skin-associated microbiomes, and **(D)** saltwater sites, Back House Point and Legendary River for intestine-associated microbiomes. Phyla with relative abundances (RA) ≥ 0.5% are shown. Phyla present at RA < 0.5% were pooled together.

Based on fish sampled at seven distinct sites on or within 200 km of King William Island, located along the lower Northwest Passage (Figure 1; Table 1), microbiome sequences were successfully obtained for 118 skin mucosal samples and 202 intestinal samples. Grouping the skin microbiome consortia by environment showed that the freshwater samples contained significantly more Shannon diversity overall than those from saline water (*R*^2^ = 0.11, *F* = 16.0, *p* < 0.05), whereas log_10_ Chao1 OTU richness was not significantly different between the two environment types (*R*^2^ = 0.00, *F* = 0.1, *p* > 0.05). In contrast, when intestinal microbiomes were grouped by environment, the saline water samples were more diverse overall than freshwater samples when log_10_ Chao1 OTU richness was considered, though this difference was small (*R*^2^ = 0.03, *F* = 4.7, *p* < 0.05). A significant difference was not observed in the intestinal microbiomes between the two environment types when Shannon diversity was considered (*R*^2^ = 0.00, *F* = 0.1, *p* > 0.05). Of the 320 skin and intestine samples combined, 60 individual Arctic char had both sets of microbiome data, allowing a comparison of skin and intestinal flora from the same fish (Figure 2). When comparing across these 60 fish, skin-associated microbiomes were significantly more diverse than intestine-associated microbiomes when both log_10_ Chao1 OTU richness (*R*^2^ = 0.60, *F* = 180.0, *p* < 0.05) and Shannon diversity (*R*^2^ = 0.52, *F* = 127.4, *p* < 0.05) were considered. In addition, though the skin and intestine communities were distinct, the skin- and intestine-associated microbiomes among individual fish appeared to be more similar at freshwater sites (Figures 2A,B) compared to saline water sites (Figures 2C,D).

The relative abundance of rarified OTUs identified to the genus level, if known, for each of the total 320 skin and intestinal microbiome samples is presented in supplemental tables (Tables S1, S2, respectively).

### Skin and Intestinal Microbiota Compositions Linked to Salinity

At freshwater sites, most taxa from skin- and intestine-associated microbiome sequence data were affiliated with Proteobacteria, but many also classified to Actinobacteria, Cyanobacteria, and Firmicutes (Figures 2A,B; Tables S1, S2). Although fish skin from saline environments was also colonized by Proteobacteria and Actinobacteria (Figure 2C), other phyla, such as Acidobacteria and Bacteroidetes, were also prominent. Intestines derived from fish sampled in saline waters were similarly characterized by Proteobacteria, Actinobacteria, and Bacteroidetes, but also by Firmicutes, Spirochaetes and Tenericutes, with considerable variation across individual fish (Figure 2D). Given these observations, depending on whether fish were sampled from fresh or saline waters, skin or intestinal bacterial communities appeared to change. For example, relative abundance based on rarified data of the psychrophile *Photobacterium* increased 200-fold in relative abundance in skin-associated microbiomes from saline sites, compared to freshwater sites (Table 2A). Similarly, relative abundance for *Deinoccoccus*, known for its ability to resist a variety of environmental stresses, increased 150-fold between fresh- and saline-caught skin microbiome fish samples. Taxa in the intestinal bacterial communities were also shown to change between fresh and saline-caught fish, with similar increases, but to a lesser magnitude including those belonging to the order Vibrionales (18-fold), the genus *Photobacterium* (14-fold), and an unknown genus in the class Mollicutes (14-fold; Table 2B).

**Table 2:** **(A)** SIMPER analysis (Primer 7) output showing relative abundances and impact ratio of environmental change between freshwater and saline locations across 118 skin-associated microbiome samples **(B)** SIMPER analysis (Primer 7) output showing relative abundances and impact ratio of environmental change between freshwater and saline locations across 202 intestine-associated samples.

**Taxonomy**	**Relative abundance**		**Impact ratio (Saline: Fresh)**
	**Saline**	**Fresh**	
**(A)**			
p: Proteobacteria c: Gammaproteobacteria o: Vibrionales f: Vibrionaceae g: *Photobacterium*	4.22	0.02	211
p: Deinococcus c: Deinococci o: Deinococcales f: Deinococcaceae g: *Deinococcus*	7.72	0.05	154
p: Proteobacteria c: Alphaproteobacteria o: Rhodospirillales f: Acetobacteraceae g: Unknown	8.58	1.52	5.64
p: Firmicutes c: Clostridia o: Clostridiales f: Clostridiaceae g: *Clostridium*	3.84	2.50	1.54
p: Proteobacteria c: Betaproteobacteria o: Burkholderiales f: Comamonadaceae g: Other	2.97	8.01	0.37
p: Proteobacteria c: Alphaproteobacteria o: Sphingomonadales f: Sphingomonadaceae g: *Sphingomonas*	1.69	5.00	0.34
p: Bacteroidetes c: Saprospirae o: Saprospirales f: Chitinophagaceae g: Unknown	1.23	4.90	0.25
p: Proteobacteria c: Alphaproteobacteria o: Rhodospirillales f: Rhodospirillaceae g: Unknown	0.90	5.69	0.16
p: Proteobacteria c: Alphaproteobacteria o: Caulobacterales f: Caulobacteraceae g: Other	0.69	4.39	0.16
p: Proteobacteria c: Betaproteobacteria o: Other f: Other g: Other	0.73	5.27	0.14
p: Proteobacteria c: Alphaproteobacteria o: Sphingomonadales f: Sphingomonadaceae g: *Novosphingobium*	0.75	6.42	0.12
p: Proteobacteria c: Gammaproteobacteria o: Xanthomonadales f: Sinobacteraceae g: Unknown	0.40	6.76	0.06
p: Proteobacteria c: Betaproteobacteria o: Burkholderiales f: Oxalobacteraceae g: *Cupriavidus*	0.12	4.90	0.02
p: Cyanobacteria c: Nostocophycideae o: Stigonematales f: Rivulariaceae g: *Rivularia*	4.80	0.00	0.00
p: Cyanobacteria c: Oscillatoriophycideae o: Oscillatoriales f: Phormidiaceae g: *Phormidium*	7.27	0.00	0.00
**(B)**			
p: Proteobacteria c: Gammaproteobacteria o: Vibrionales f: Other g: Other	3.10	0.17	18.23
p: Tenericutes c: Mollicutes o: Unknown f: Unknown g: Unknown	7.32	0.52	14.08
p: Proteobacteria c: Gammaproteobacteria o: Vibrionales f: Vibrionaceae g: *Photobacterium*	20.8	1.48	14.03
p: Spirochaetes c: Brevinematae o: Brevinematales f: Brevinemataceae g: Unknown	4.54	0.54	8.41
p: Proteobacteria c: Gammaproteobacteria o: Vibrionales f: Vibrionaceae g: Other	6.06	1.09	5.56
p: Proteobacteria c: Gammaproteobacteria o: Vibrionales f: Vibrionaceae g: *Aliivibrio*	5.08	1.22	4.16
p: Proteobacteria c: Alphaproteobacteria o: Sphingomonadales f: Sphingomonadaceae g: *Sphingomonas*	5.91	4.06	1.46
p: Tenericutes c: Mollicutes o: Mycoplasmatales f: Mycoplasmataceae g: *Mycoplasma*	4.17	4.42	0.94
p: Fusobacteria c: Fusobacteriia o: Fusobacteriales f: Fusobacteriaceae g: *u114*	2.77	4.12	0.67
p: Proteobacteria c: Gammaproteobacteria o: Pseudomonadales f: Pseudomonadaceae g: *Pseudomonas*	1.65	3.50	0.47
p: Proteobacteria c: Betaproteobacteria o: Burkholderiales f: Comamonadaceae g: Other	2.50	5.96	0.42
p: Proteobacteria c: Gammaproteobacteria o: Pseudomonadales f: Pseudomonadaceae g: Other	2.23	7.91	0.28
Unclassified Taxa	1.23	7.65	0.16
p: Proteobacteria c: Alphaproteobacteria o: Caulobacterales f: Caulobacteraceae g: *Phenylobacterium*	0.40	5.71	0.07
p: Proteobacteria c: Betaproteobacteria o: Neisseriales f: Neisseriaceae g: *Deefgea*	0.05	2.86	0.02

*Taxonomy from phylum (p) to clade (c) to order (o) to family (f) to genus (g) is shown*.

**Figure 3:**
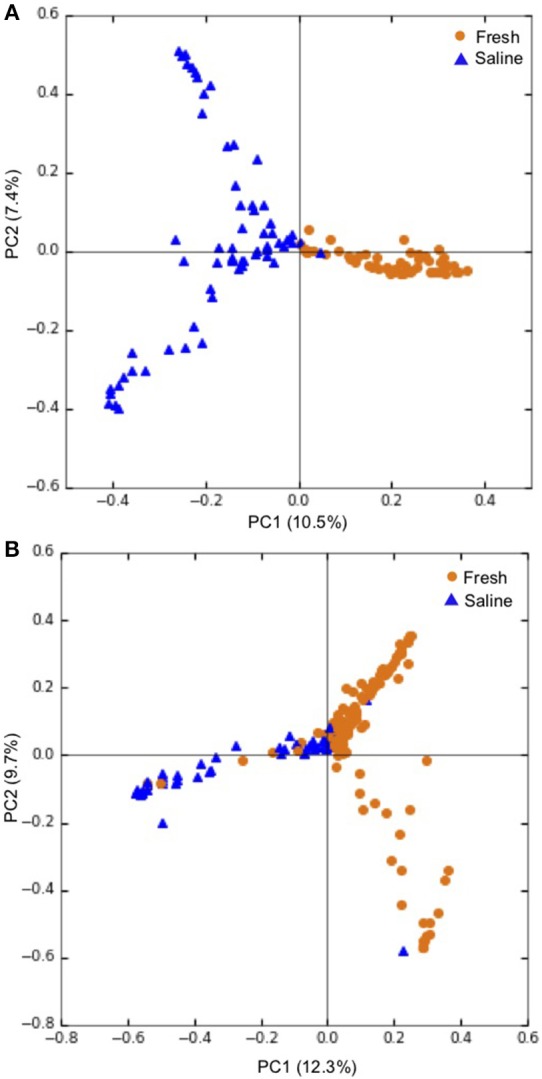
Principal coordinate analysis (PCoA) showing grouping of **(A)** individual skin microbiomes at fresh (*n* = 55), and saline (*n* = 63) sites and **(B)** individual intestinal microbiomes at fresh (*n* = 144) and saline (*n* = 58) sites. PCoA ordinations are based on the Bray-Curtis dissimilarity metric. Freshwater sites include lake and river sites sampled in winter and spring while saline sites refer to sea shoreline locations sampled in autumn during the annual char run.

**Figure 4:**
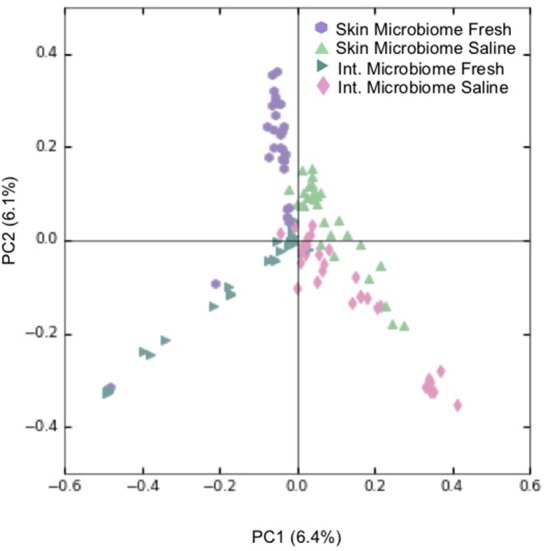
Principal coordinate analysis (PCoA) showing clustering of individual skin and intestinal microbiome communities based on Bray-Curtis dissimilarity. For every skin-associated community represented, a counterpart intestine-associated community from the same *S. alpinus* individual is shown. Legend refers to the sample type and environment of where an *S. alpinus* individual was caught and includes skin microbiomes from freshwater sites (*n* = 30), and from saline sites (*n* = 30), as well as intestinal microbiomes from freshwater sites (*n* = 30) and from saline sites (*n* = 30). Fresh refers to lake and river sites sampled in winter and spring while saline refers to sea shoreline locations sampled in autumn during the annual char run.

Overall, PCoA combined with ANOSIM showed a statistically significant separation between samples obtained from freshwater and saline sites for both skin microbiomes (*p* < 0.001; Figure 3A), and intestinal microbiomes (*p* < 0.001; Figure 3B). This indicates that salinity is a primary factor in defining these microbial communities. For the 60 skin and intestinal microbiome samples used for within-fish comparisons where possible, ANOSIM showed that the communities obtained from the two sample types were significantly different (*p* < 0.001; Figure 4).

In addition, preliminary results show that the community structure of identified bacteria from water samples at sites corresponding to where fish were caught (excluding Back River) differs from that of the fish, suggesting that the distribution of microbiota identified on the fish are influenced by physiological processes inherent to the fish (Figure S1). However, the phyla Proteobacteria, Bacteroidetes, and Actinobacteria are represented in both the water microbiomes and in both skin- and intestine-associated microbiota. Across water samples, no significant difference was observed between freshwater and saline sites overall for either Chao1 OTU richness (*R*^2^ = 0.01, *F* = 0.2, *p* > 0.05), or Shannon diversity (*R*^2^ = 0.07, *F* = 1.6, *p* > 0.05). The most similarity was observed between samples from similar geographic regions. For example, Port Perry, Swan Lake, and Koka Lake were from freshwater sites on King William Island whereas the saline sites Back House Point, Legendary River, and the freshwater site Murchison River were from Chantrey Inlet.

### Characterization of the Skin Microbiota

**Figure 5:**
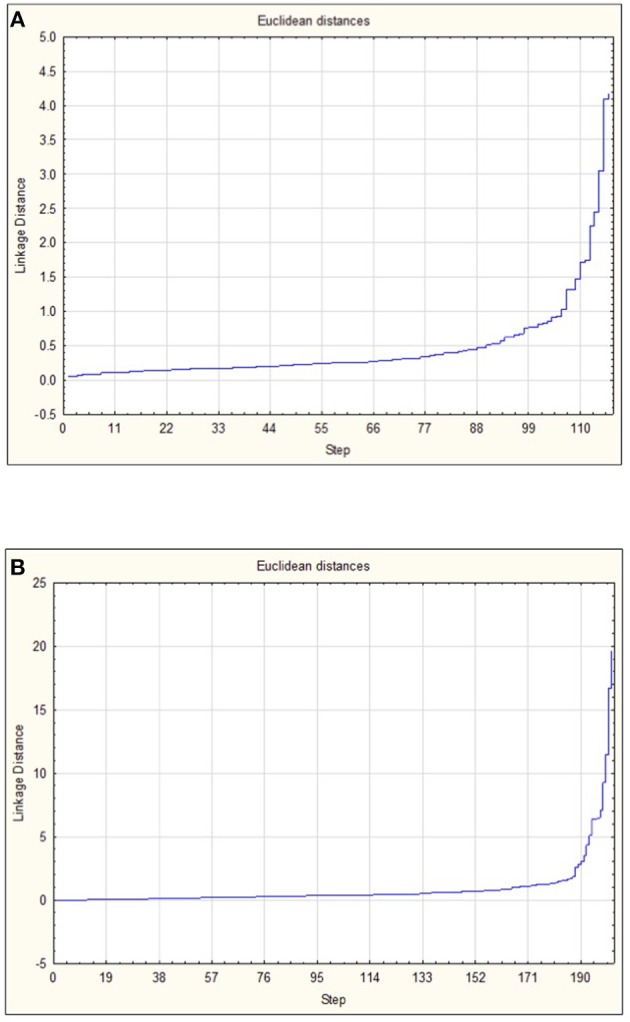
Amalgamation schedule used to identify the number of major clusters (K) for **(A)** OTUs from skin-associated microbiomes and **(B)** OTUs from intestine-associated microbiomes. Linkage distance is shown in addition to the visualization of steps.

When the 118 skin-associated microbiome samples were analyzed using hierarchical Ward's linkage clustering (Dillner et al., 2005), eight distinct clusters were apparent in the amalgamation schedule graph (Figure 5A), with the decreasing linkage distance after the seventh fusion step indicating a minimal difference for any newly formed clusters. Euclidean distances between the centers of clusters confirmed the distinct nature of each cluster, with all clusters largely apart from each other (Table S3). Subsequent K-means clustering was then carried out to identify members of each of the clusters (Table 3A). The largest cluster (#6) contained 98% of the freshwater (54/55) and 52% of the saline (32/62) samples, supporting the distinct grouping of saline and freshwater samples observed in the PCoA ordinations (Figure 3). Because many of the fish were netted in autumn, during the annual char migration, it is probable that some saline-associated microbiota could remain in the same cluster as the microbiota from freshwater fish. The ANOVA results show that of the 899 taxa used in the clustering analysis, 56 have a *p*-value below 0.05. These results also indicated that Proteobacteria, Cyanobacteria, and Firmicutes contributed most to the clustering analysis (Table S4). As an approach to identify a core microbiome for Arctic char skin, bacteria present across the eight clusters were identified (Table 4A). The fact that none of the nine taxa, except for *Clostridium*, matched to any known genus likely emphasizes the paucity of research for this wild species. For example, an unknown genus from the Acetobacteraceae family represents over 5% of the total microbiota. In contrast to the clustering based on a saline environment, no correlation was obtained between the presence of any of the microbial genus species and the sex, age, or size of the Arctic char samples (*p* > 0.05; data not shown).

**Table 3:** **(A)** Sample counts of each cluster, showing number of samples either caught in a saline or freshwater environment, as obtained using K mean clustering analysis using the skin microbiome data at genus level and **(B)** Sample counts of each cluster, showing number of samples either caught in a saline or fresh environment, as obtained using K mean clustering analysis using the intestinal microbiome data at genus level.

**Cluster**	**Saline**	**Fresh**
**(A)**		
1	9	0
2	12	0
3	3	0
4	1	1
5	1	0
6	31	54
7	1	0
8	3	0
**(B)**		
1	2	7
2	0	8
3	1	11
4	21	82
5	0	14
6	19	2
7	1	19
8	8	1

**Table 4:** **(A)** Microorganisms, classified to genus where possible, present in eight clusters along with the percentage within the skin microbiome of Arctic char and **(B)** Microorganisms, classified to genus where possible, present in eight clusters along with the percentage within the intestinal microbiome of Arctic char.

**Taxonomy**	**Cluster number and percent abundance**							
	**1**	**2**	**3**	**4**	**5**	**6**	**7**	**8**
**(A)**								
Other bacteria	0.96	1.61	2.04	0.28	0.52	1.93	0.81	0.74
p: Actinobacteria c: Actinobacteria o: Actinomycetales f: Other g: Other	0.30	0.81	0.85	0.05	0.19	0.80	0.05	0.30
p: Proteobacteria c: Other o: Other f: Other g: Other	0.07	0.20	0.03	0.17	0.09	0.55	0.05	0.03
p: Proteobacteria c: Alphaproteobacteria o: Rhodospirillales f: Acetobacteraceae g: Unknown	0.45	4.14	4.73	0.05	1.52	5.15	0.14	0.03
p: Cyanobacteria c: Other o: Other f: Other g: Other	0.41	3.99	0.19	0.00	0.19	0.32	0.05	0.06
p: Firmicutes c: Bacilli o: Lactobacillales f: Other g: Other	2.67	0.20	0.02	0.02	0.00	0.17	3.94	57.1
p: Firmicutes c: Clostridia o: Clostridiales f: Clostridiaceae g: *Clostridium*	2.42	0.22	72.0	0.00	0.14	0.78	0.09	0.27
p: Proteobacteria c: Alphaproteobacteria o: Rhodobacterales f: Rhodobacteraceae g: Other	0.11	0.30	0.14	0.02	70.2	0.94	0.00	0.09
p: Proteobacteria c: Betaproteobacteria o: Burkholderiales f: Comamonadaceae g: Other	0.13	0.21	0.08	87.42	0.47	2.73	0.00	0.03
**(B)**								
Unclassified	2.16	2.17	1.48	8.20	6.26	0.21	1.28	0.15
Other Bacteria	0.39	0.38	3.62	1.37	1.03	0.38	0.16	1.09
p: Actinobacteria c: Actinobacteria o: Actinomycetales f: Other g: Other	3.59	0.01	0.02	0.14	0.01	0.03	0.05	0.16
p: Firmicutes c: Bacilli o: Lactobacillales f: Streptococcaceae g: *Streptococcus*	0.03	0.28	1.02	2.35	1.81	0.08	0.34	0.44
p: Fusobacteria c: Fusobacteriia o: Fusobacteriales f: Fusobacteriaceae g: *u114*	0.03	0.04	84.2	0.79	0.02	1.64	0.01	2.70
p: Proteobacteria c: Alphaproteobacteria o: Sphingomonadales f: Sphingomonadaceae g: *Sphingomonas*	9.56	0.02	0.17	10.5	0.23	0.62	0.06	0.46
p: Proteobacteria c: Betaproteobacteria o: Burkholderiales f: Comamonadaceae g: Other	0.07	0.11	0.01	2.48	52.5	0.61	1.01	0.17
p: Proteobacteria c: Gammaproteobacteria o: Alteromonadales f: Shewanellaceae g: *Shewanella*	2.74	0.57	0.03	1.29	1.50	0.57	0.04	0.03
p: Proteobacteria c: Gammaproteobacteria o: Pseudomonadales f: Pseudomonadaceae g: Other	0.08	8.23	2.46	1.27	1.32	0.45	85.0	0.20
p: Proteobacteria c: Gammaproteobacteria o: Pseudomonadales f: Pseudomonadaceae g: *Pseudomonas*	0.01	0.70	0.14	2.66	1.47	0.31	3.80	0.15
p: Proteobacteria c: Gammaproteobacteria o: Vibrionales f: Vibrionaceae g: Other	0.11	0.01	0.17	2.43	0.47	6.11	0.01	0.09
p: Proteobacteria c: Gammaproteobacteria o: Vibrionales f: Vibrionaceae g: *Aliivibrio*	0.20	0.01	0.13	3.20	0.80	3.47	0.01	0.04
p: Proteobacteria c: Gammaproteobacteria o: Vibrionales f: Vibrionaceae g: *Photobacterium*	0.24	0.27	0.68	1.78	0.02	71.0	0.38	4.49
p: Tenericutes c: Mollicutes o: Mycoplasmatales f: Mycoplasmataceae g: *Mycoplasma*	73.8	0.20	1.37	2.11	0.36	1.19	0.42	2.14

*Taxonomy from phylum (p) to clade (c) to order (o) to family (f) to genus (g) is shown*.

### Characterization of the Intestinal Microbiota

The 202 intestinal microbiome samples were tested by hierarchical Ward's linkage clustering (Dillner et al., 2005) to investigate the link between salinity and intestine-associated microbiota. The amalgamation schedule graph again showed seven major fusion steps, resulting in eight distinct clusters (Figure 5B) with Euclidean distances between clusters showing their distinctive nature (Table S5). Of the 59 intestinal microbiome samples from freshwater and 143 from saline sites, two clusters (#6 and #8) were dominated by samples from saline water sites whereas another two clusters (#2 and #5) only showed samples from freshwater (Table 3B). Therefore, these clusters provide further support to the PCoA analysis (Figure 3) that salinity is a statistically significant variable defining the Arctic char intestine-associated microbiome. The ANOVA results indicate that of the 507 taxa used in the clustering analysis, 50 have a *p*-value below 0.05. The phylum Proteobacteria accounted for 25 of these taxa (Table S6). Members defined as belonging to an “unidentified kingdom” represented over 6% of the microbial community in two clusters (#4 and #5). Overall, the intestinal core microbiome appears more diverse than that of the skin, with 14 taxa present in all eight clusters (Table 4B). Clusters (#1–#7) included taxa that represented ~8% or more of the community, with 4 and 8 clusters with a single genus occupying over 50% of the entire intestinal microbiome. The taxa included unidentified genera from families Fusobacteriaceae, Comamonadaceae, Pseudomonadaceae, and Vibrionaceae. Other members of the intestinal-associated microbiome include *Streptococcus, Sphingomonas, Shewanella, Pseudomonas, Aliivibrio, Photobacterium*, and *Mycoplasma* (Table 4B). Again, no correlation was obtained between the presence of any of the microbial genus species and the sex, age, or size of the Arctic char (*p* > 0.05; not shown). Additionally, since hierarchical Ward's linkage clustering was implemented on skin and intestine samples without regard to fishing site, we postulate that the following results provide further evidence that the changes in microbiome between fish caught in saline and freshwater environments are strongly linked to salinity.

## Discussion

### Microbial Structure in the Skin and Intestinal Mucous

The commensal microbes contributing to these Arctic char skin- and intestine-associated microbiome communities were found to be distinct from one another (Table 2; Figure 2; Tables S1, S2). There have been few published reports on skin-associated microbiomes, especially in wild fish, possibly because of the challenges associated with ensuring aseptic collection practices and optimal preservation techniques (Kueneman et al., 2014). Microbiome analysis on the skin of anadromous Atlantic salmon, *S. salar*, showed that Proteobacteria was the dominant phylum (Lokesh and Kiron, 2016), and it was also dominant in the majority of our skin samples taken from freshwater Arctic char (Figure 2A). Significant differences in the microbiome from fish caught in fresh and saline waters suggests that the skin microbial community changes as the fish swim from one environment to another. Part of the challenge, and likely reflecting the generally unstudied field, is the large proportion of unknown genera in the skin-associated microbiome; 8 of 9 organisms making up the putative core microbiome are currently unknown, and in addition, over 1% of the microbial community belonged to unknown phyla (Table 4A). Some of these currently unknown members likely serve important roles in the skin mucous, suggesting that metagenomic analysis could perhaps be used to assign putative roles in waste removal, osmoregulation, glycoprotein-mediated drag reduction, and in immune function, and would be of interest for future experiments.

In contrast to the paucity of information on skin-associated microbiomes, intestine-associated microbiomes have been better studied, although not extensively in wild, ocean-going fish (Egerton et al., 2018). Previously reported microorganisms present in the Arctic char gut include *Aeromonas, Flavobacterium, Pseudomonas, Lactobacillus*, and *Vibrio* (Ringø and Strøm, 1994; Ringø et al., 1995; Nyman et al., 2017). Lactic acid bacteria (phylum Firmicutes), Fusobacteria, Bacteroidetes, Gammaproteobacteria, Planctomycetes, Clostridia, Verrumicrobia and Bacilli have all been reported as a normal part of the intestine-associated microbiomes in several fish species (Ringø and Gatesoupe, 1998; Befring-Hovda, 2007; Ingerslev et al., 2014; Ghanbari et al., 2015). The results reported here are therefore consistent with the previous literature, demonstrating that the community found in wild adult *S. alpinus* intestine-associated microbiomes is not fundamentally different from other fish.

The core intestine-associated microbiome in Arctic char includes taxa from the Gammaproteobacteria class as well as three genera within the Vibrionaceae family (Table 4B), similar to observations from Antarctic notothenioid fish species (Ward et al., 2009). We also noted the presence of *Streptococcus* and *Mycoplasma. Mycoplasma* have been reportedly abundant in Antarctic fish and Atlantic salmon (Holben et al., 2002; Song et al., 2016; Dehler et al., 2017). Unlike in humans where *Mycoplasma* and certain *Streptococcus* are associated with dysbiosis, these genera are proposed to be important for lipid and sugar metabolism and are therefore included in the core intestinal microbiome for Atlantic salmon (Dehler et al., 2017). In vertebrates, *Sphingomonas* have been associated with the early-life education of the immune system (Olszak et al., 2012; Wingender et al., 2012; Caballero and Pamer, 2015; Gensollen et al., 2016), and therefore may play a similar role in the gut of Arctic char.

Notwithstanding the distinct profiles of the skin- and intestine-associated microbiomes, they appear to share certain taxa, including psychrophilic genera, such as *Psychrobacter, Shewanella, Flavobacterium, Acinetobacter, Photobacterium, Planctomyces*, and *Psychromonas*, and including bacteria belonging to the phyla Firmicutes and Cyanobacteria, which could contribute to geochemical cycles, including the nitrogen cycle. Certainly, both microbial community compositions were significantly influenced by the environment in which the fish were caught (Figure 3; Table 3), with *Photobacterium* and *Deinococcus* increasing over 1–2 orders of magnitude when intestine- and skin-associated microbiomes, respectively, were recovered from saline water samples. There was no apparent correlation with other measured biotic factors. Our results on wild Arctic char populations are in accord with previous reports including Atlantic salmon, in that they showed salinity-mediated turnover in microbial distributions across the skin and intestines (Schmidt et al., 2015; Lokesh and Kiron, 2016; Dehler et al., 2017). Manipulation of the skin and intestinal microbiomes by deliberate salinity changes in aquaculture has not yet been explored, but our results suggest that a directed turnover in taxa might be achieved by such a protocol, and should be considered to prevent or inhibit dysbiosis. Although there has been some examination of the survival of farmed *S. alpinus* at higher salinities, consensus has not yet been reached on best practices for inland recirculating systems (Jørgensen et al., 1993; Larsson and Berglund, 1998; Summerfelt et al., 2004; Duston et al., 2007). To date, existing aquaculture studies have focused on the osmoregulatory consequences of keeping stocks at low salinity or the transfer of young fish from freshwater to a potential saltwater grow-out phase (Arnesen et al., 1993; Aarset, 1999; Duston et al., 2007), but there has been little consideration of the Arctic char skin-associated microbiome, nor of skin- and intestine-associated microbiomes together, and yet the biotechnological applications for manipulation of the microbiota by probiotics is of considerable interest.

### Bioprospecting in the Skin and Intestinal Microbiomes

Anadromous Arctic char from this high Arctic region grow more rapidly than other local salmonids and live several decades (McPhedran et al., unpublished data). Therefore, we posit that knowledge of the microbiota could be helpful in the development of sustainable and efficient aquaculture practices and to provide an alternate modality to overcome the adverse effects of antibiotics and drugs (Nayak, 2010). As suggested above, consideration could be given to the manipulation of salt concentrations in Arctic char aquaculture to deliberately direct bacterial communities and possibly bypass dysbiotic episodes. Alternatively, individual isolates may prove valuable. Previously, lactic acid bacteria including *Streptococcus* have been used as probiotics and specifically for furunculosis in rainbow trout, *Oncorhynchus mykiss* (Balcázar et al., 2007; Pandiyan et al., 2013). This is a serious infection of farmed fish causing skin lesions, hemorrhaging, intestinal tissue damage, and death (Ringø et al., 2004). Other studies have reported the beneficial effect of *Shewanella* and *Vibrio* probiotics (Kamei et al., 1988; Irianto and Austin, 2002; Díaz-Rosales et al., 2006a,b). The bacteria that we detected in Arctic char skin- and intestine-associated microbiomes, especially those identified as part of the core microbiome, therefore have potential in probiotic applications for aquaculture where they could play a role in immune development and promote the health and growth of farmed fish.

Many OTUs in the skin and intestinal microbiomes represented unidentified microorganisms, and it suggests that the microbial diversity associated with these wild Arctic char provides fertile ground for bioprospecting for psychrophiles and osmotolerant organisms. For example, some known organisms that are psychrophilic and halophilic are a good source for polyunsaturated fatty acids (PUFA) (Russell and Nichols, 1999). Russell (1998) proposed that PUFA in marine psychrophiles allows them to balance the requirement for a fluid membrane at low temperatures with the retention of a requisite level of order. Given these functions, PUFA-producing bacteria may represent an alternate source for human use, with the added advantage of containing only one long-chain PUFA rather than the multiple components present in fish or algal oils (de Pascale et al., 2012).

As indicated, taxa involved in nitrogen fixation (Cyanobacteria and Rhizobiales), nitrate oxidation (Nitrospirales), and nitrite oxidization (*Nitrospira*) are present in the skin- and intestine-associated microbiomes (Tables S1, S2). Their presence suggests that they may provide an inorganic source of nitrogen when organic nitrogen is low, such as when prey may not be as readily available during the winter under ice. Similar results have been observed by Lee and Childress (1994) and Shah et al. (in press) in marine invertebrates. Isolates of Nitrospirales, *Nitrospira*, and *Paracoccus*, identified in Arctic char that are adapted to low temperature conditions might be employed in municipal and domestic wastewater treatment facilities where wastewater temperatures fall below 10 °C in winter, and when microbial activity is normally severely depressed (Xu et al., 2018). Similarly, isolates of *Pseudomonas, Shewanella, Bacillus, Arthrobacter*, and *Sphingobacterium* in the intestine-associated microbiome (Table 4B) could play an important role in degradation of pollutants during wastewater treatment. Some bacteria (e.g., members of the Rhizobiales) could even find utility as nitrogen fixers if used for agricultural applications under low temperature conditions.

Bioprospecting need not be restricted to probiotics, however. Further exploration of identified extremophiles may be useful for other applications. For example, between 6 and 8 million tons of waste crab, shrimp, and lobster shells are produced globally, with the chitin-rich wastes dumped in landfills or the sea (Yan and Chen, 2015). The presence of OTUs representing *Photobacterium*, in the intestine-associated microbiome of Arctic char (Table 2B), with members of this taxon known to aid in chitin digestion (MacDonald et al., 1986; Ramesh and Venugopalan, 1989; Itoi et al., 2006) is noteworthy. It suggests that in the future, chitin-containing seafood waste could be used as supplements for fish food in aquaculture facilities, and also used as a probiotic supplement for Arctic char and other fish.

In general, our results highlight the need to further explore structural and functional aspects of the microbial communities that naturally inhabit *S. alpinus*. The turnover of both the skin- and intestine-associated communities together during migration represents a previously underappreciated stressor for the species that could be exploited for biotechnological applications including advanced aquaculture systems. The specific taxa observed suggest the potential for isolating useful probiotics, whilst the number of undefined taxa exposes a lack of understanding of this ecosystem, the resolution of which could benefit our understanding of Arctic marine ecosystems as a whole.

## Data Availability

The datasets generated for this study can be found in European Nucleotide Archive, ERP111452.

## Ethics Statement

Fish were sampled in accordance with an approved and issued license to fish for scientific purposes in the waters of the Northwest Territories, Yukon north slope, and Nunavut (in accordance with section 52 of the general fishery regulations of the fisheries act, Fisheries and Oceans, Canada; current permit number S-18/19-1045-NU) along with an associated animal care permit approved and issued by the Fresh Water Institute Animal Care Committee of the Department of Fisheries and Oceans (current permit number FWI-ACC AUP-2018-63).

## Author Contributions

PvCdG organized the fish collection. JDN and KE undertook sequencing and provided bioinformatics analysis. EFH and GE isolated the DNA and conducted statistical analyses. VS did additional statistical analyses. VKW designed the study. EFH, VS, and VKW drafted the manuscript. All authors have read and accepted the final manuscript.

### Conflict of Interest Statement

The authors declare that the research was conducted in the absence of any commercial or financial relationships that could be construed as a potential conflict of interest.
