# Supplementary material for: Anadromous Arctic Char Microbiomes: Bioprospecting in the High Arctic
Source: Front Bioeng Biotechnol. 2019 Feb 26;7:32. doi: 10.3389/fbioe.2019.00032 (PMC6399304; doi:10.3389/fbioe.2019.00032)
Supplement: Supplementary file 3 [file Table_3.pdf]

Table S3: Euclidean distances between the eight clusters based on the microbiota present in the skin of arctic char.

	Cluster 1	Cluster 2	Cluster 3	Cluster 4	Cluster 5	Cluster 6	Cluster 7	Cluster 8
Cluster 1	0.00							
Cluster 2	0.02	0.00						
Cluster 3	0.03	0.03	0.00					
Cluster 4	0.03	0.03	0.04	0.00				
Cluster 5	0.03	0.03	0.03	0.04	0.00			
Cluster 6	0.01	0.01	0.02	0.03	0.02	0.00		
Cluster 7	0.02	0.02	0.03	0.04	0.03	0.02	0.00	
Cluster 8	0.02	0.02	0.03	0.04	0.03	0.02	0.03	0.00
