# Supplementary material for: Anadromous Arctic Char Microbiomes: Bioprospecting in the High Arctic
Source: Front Bioeng Biotechnol. 2019 Feb 26;7:32. doi: 10.3389/fbioe.2019.00032 (PMC6399304; doi:10.3389/fbioe.2019.00032)
Supplement: Supplementary file 7 [file Image_1.pdf]

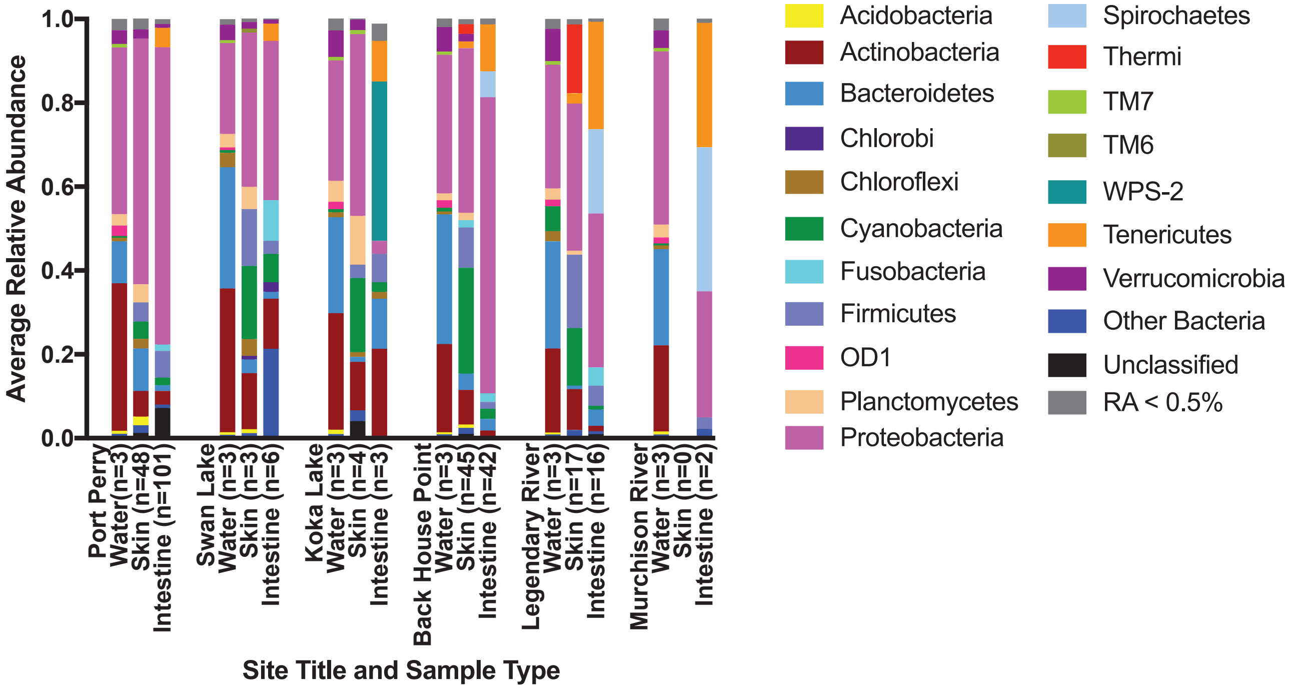

Figure S1: Representation of average relative abundance across all samples identified in both the water corresponding to where fish were caught, as well as corresponding fish and intestinal microbiomes at each site (excluding Back River). Phyla with relative abundances (RA) ≥ 0.5% are shown. Phyla present at RA < 0.5% were pooled together. Water samples were collected in triplicate and filtered on-site, while filters were frozen at -20 ºC, transported, moved to -80 ºC for storage, and analyzed through PCR amplification on an Illumina sequencing platform with primers 515-F and 806-R. Note that the primers and analysis pipeline for water differs from that of skin and intestinal microbiomes, rendering these results as preliminary.

frontiers
